# Supplementary material for: A scalable platform for acquisition of high-fidelity human intracranial EEG with minimal clinical burden
Source: PLoS One. 2024 Jun 13;19(6):e0305009. doi: 10.1371/journal.pone.0305009 (PMC11175507; doi:10.1371/journal.pone.0305009)
Supplement: S1 Appendix — These settings work against a fresh device reset of a mikrotik router and are used to provision both server and client routers. Default mikrotik IP is 192.168.88.1. (PDF) [file pone.0305009.s001.pdf]

Appendix

```
/system identity set name=<mikrotik name>
/ip dhcp-server disable 0
/ip dhcp-client disable 0
/interface bridge port remove 0
/ip address add address=<ip address> netmask=<mask address> interface=ether1
/ip route add gateway=<gateway address>
/ip ipsec proposal set 0 auth-algorithms=sha512
/interface eoip add name=<eoip name> tunnel-id=0 mac-address=<mac address>
    mtu=1500 remote-address=<remote address> ipsec-secret=<secret> allow-fast-path=no
/interface bridge add name=eoip-bridge mtu=1500 protocol-mode=none
/interface bridge port add bridge=eoip-bridge interface=<eoip name>
/interface bridge port add bridge=eoip-bridge interface=ether2
```

S1 Appendix. Configuration commands for router provisioning. These settings work against a fresh device reset of a mikrotik router and are used to provision both server and client routers. Default mikrotik IP is 192.168.88.1.
