# Supplementary material for: A scalable platform for acquisition of high-fidelity human intracranial EEG with minimal clinical burden
Source: PLoS One. 2024 Jun 13;19(6):e0305009. doi: 10.1371/journal.pone.0305009 (PMC11175507; doi:10.1371/journal.pone.0305009)
Supplement: Attachment — Submitted filename: 240204_21_ieeg_infrastructure_plos_one_response.pdf [file pone.0305009.s003.pdf]

Response to Reviewers

Article reference: PONE-D-23-31649

Authors' Thank You

We thank the academic editor and reviewers for their time and attention to this work. We were delighted to hear that both reviewers found the work well motivated and compelling. Please find our response below.

Academic editor/reviewer comments appear in **black**, author responses appear in **blue**, and changes to the manuscript in response to academic editor/reviewer comments appear in **red**.

Academic editor

I believe that the paper is generally well-written. However, both reviewers have mentioned the need for revisions, and I agree that modifications are necessary. Therefore, I kindly request that you make the necessary changes in accordance with the comments of the two reviewers.

Thank you for your interest in our work. We appreciate both reviewers' comments and made changes to our manuscript accordingly. We have also reviewed the journal requirements and addressed any necessary changes.

Reviewer 1

Dr. Yamada et al. reported a methodology and system for recording intracranial EEG data of drug-resistant epilepsy patients' evaluations, with 10kHz iEEG up to 256 electrodes simultaneously, along with clinical data. As mentioned in the manuscript, higher-fidelity iEEG has merits in accurately localizing the epileptogenic zone and using the data for understanding physiological human neuroscience. In this context, their suggested methodology offers minimal invasiveness to clinical courses and patients, as well as higher temporal resolution data. The authors described the background in the introduction and the flow of intracranial EEG data with clinical burden, as shown in Figure 1. This makes it easier for readers who are not familiar with the detailed methodology to understand the principles of their suggested methodology and system. Their description in the introduction and method not only justifies their research and emphasizes its clinical impact but also makes the manuscript easy to follow.

We thank the reviewer for recognizing the importance of the work and providing an appropriate summary of the manuscript.

However, my concern with publishing this article as a "Research Article" is the lack of statistical comparison with a control. Generally speaking, methodology papers are required to be compared with the conventional method to demonstrate their validity as a new method or system. Therefore, I suggest the authors consider either of the following options:
(1) Submitting as "Submissions describing methods, software, databases, or other tools." Please find the details of the article types in PLOS ONE:
`https://journals.plos.org/plosone/s/journal-information#loc-scope`.
(2) Attaching data that shows the validity of the suggested methodology or system with a comparison to the conventional method. Additionally, I suggest describing the weaknesses of this method. For example, 10kHz recording holds ten times more information than 1kHz, which means it requires ten times more data space and longer analysis time.

We appreciate the suggestion. We plan to submit this manuscript as "Submissions describing methods, software, databases, or other tools." A comparison of various methodologies, describing their advantages and disadvantages, can be found in "Research EEG acquisition approach." We have added a sen-

tence in the Introduction to acknowledge that the higher sampling rate will result in larger datasets and refer to "Scalable solutions for large (> 1 TB) datasets," which discusses our approach for handling these large datasets.

Page 3, line 37:
While a higher sampling rate requires more resources for data processing, data management tools (described in Scalable solutions for large (> 1 TB) datasets) can help facilitate the analysis and storage of these datasets.

Reviewer 2

Yamada et al. reported their technical approach for building comprehensive data repositories of more than 200 participants of diverse demographics. They mentioned that their promising approach is promoting equitable enrollment and building comprehensive data repositories with consistent, high-fidelity specifications towards new discoveries in human neuroscience.

Comments (invitation: November 28, 2023, and submission: November 29, 2023)

1) We want to congratulate the authors' efforts. The manuscript is well written and easy to follow. By establishing a research platform that minimizes clinical burden, it becomes feasible to record data from a substantial number of participants while maintaining consistency. None of the following comments are criticisms.

We thank the reviewer for acknowledging the effort put into deploying this research platform and its potential impact on clinical studies.

2) Introduction: Would it be possible to provide the reasons behind the acquisition of 10 kHz iEEG data? The authors mentioned in this article that 5 kHz is recommended (line 34). It would be nicer to include the authors' perspective on this matter in the Discussion section (High-quality iEEG data repository subsection).

Certain studies recommend 5 kHz for high frequency oscillation analyses;

however, it is still unclear if even higher sampling rates would be beneficial. Therefore, we are recording from the highest sampling rate supported by our hospital-integrated platform. We have re-emphasized this in numerous parts of the manuscript, including the Discussion as suggested.

Page 3, line 33:
It still remains a question about how fast these oscillations can reach [15,16]; thus, this work aimed to collect iEEG with the highest sampling rate supported by our hospital-integrated platform, 10 kHz, enabling a full range of temporal activity for quantitative analyses.

Page 7, line 146:
This allows the secondary research stream to sample neuroelectrophysiology at 10 kHz (i.e., the highest supported sampling rate on the JE-120A) independently and simultaneously with the clinical data stream using the same acquisition system.

Page 17, line 408:
This is the highest sampling rate supported by the clinical system, enabling a small research footprint in the patient room.

3) Methods and Figure 1: Would it be possible to specify which stage in Figure 1 corresponds to the method employed in this study? Figure 1, as described by the authors, delineates five distinct stages to obtaining and storing clinical EEG data (line 53 and 54), with each stage elaborated on in the methods section. It would be nicer to provide a visual representation within Figure 1 to highlight the method used by the authors.

The research digital stream method is employed in this study. This was mentioned in the caption of Figure 2 (originally Figure 1) and the text in "Research EEG acquisition approach," but we have further visually emphasized this in the figure by greying out the other research acquisition methods that were not used. The figure caption was also updated to reflect this.

4) Methods and Figure 2: To explain "the integration of our research data infrastructure in a hospital setting" (line122 and 123), Figure 2 is being used; however, I wondered if Figure 2 might not be necessary for this paper. If it is to be used, adding some explanation on how the integration leads to the

generation of Figure 2 would make it more understandable for readers.

Figure 1 (originally Figure 2) may not have much educational value for those deeply in the field, but we believe it is useful for readers who may not be familiar with intracranial EEG studies. We have moved the figure to the Introduction, where we feel it may be more appropriate and revised the contents of the figure.

5) Results: Would it be possible to provide the difference in the number of research papers produced at the authors' facility before and after the actual use of this platform. It would be helpful for readers to understand the difference.

This platform enables unique possibilities mentioned in "Opportunities for human neuroscience" and describes past work using this data repository ([58], [59]). These papers (and additional pending work) would not have been published otherwise.

6) Figure 1, 3 and Table 1: It would be nicer to include the explanations of the abbreviations in the legend.

Thank you, we have added some notes on abbreviations for each caption of Figure 1, 3, and Table 1.

Journal Requirements

When submitting your revision, we need you to address these additional requirements.

1. Please ensure that your manuscript meets PLOS ONE's style requirements, including those for file naming. The PLOS ONE style templates can be found at https://journals.plos.org/plosone/s/file?id=wjVg/PLOSOne_formatting_sample_main_body.pdf and https://journals.plos.org/plosone/s/file?id=ba62/PLOSOne_formatting_sample_title_authors_affiliations.pdf

We have reviewed the PLOS ONE's style requirements and updated our manuscript accordingly.

2. Thank you for stating in your Funding Statement: "PN received support from Stanford Wu Tsai Neurosciences Institute and Stanford Bio-X Seed Grant IIP-9 104."
Please provide an amended statement that declares *all* the funding or sources of support (whether external or internal to your organization) received during this study, as detailed online in our guide for authors at http://journals.plos.org/plosone/s/submit-now. Please also include the statement "There was no additional external funding received for this study." in your updated Funding Statement. Please include your amended Funding Statement within your cover letter. We will change the online submission form on your behalf.

We have updated our funding statement and included it in our revised cover letter.

Page 20, line 495:
This work is supported by National Institute of Health (NIH) U19NS118284, Stanford Bio-X Seed Grant IIP9-104, and the Stanford Wu Tsai Neurosciences Institute. There was no additional external funding received for this study.

3. One of the noted authors is a group or consortium Stanford Comprehen-

sive Epilepsy Center and Stanford Pediatric Epilepsy Center. In addition to naming the author group, please list the individual authors and affiliations within this group in the acknowledgments section of your manuscript. Please also indicate clearly a lead author for this group along with a contact email address.

We moved group member information to the Acknowledgments and updated it with individual group membership enumeration. The corresponding author's name and email address are denoted with * in the authors and affiliation section.

Page 21, line 507:
Authorship and non-author group contributors followed the recommendations of the International Committee of Medical Journal Editors (ICMJE) and Council of Science Editors (CSE).

(Individual group membership enumeration in manuscript.)

4. We note that Figures 3 and 4 in your submission contain copyrighted images. All PLOS content is published under the Creative Commons Attribution License (CC BY 4.0), which means that the manuscript, images, and Supporting Information files will be freely available online, and any third party is permitted to access, download, copy, distribute, and use these materials in any way, even commercially, with proper attribution. For more information, see our copyright guidelines: `http://journals.plos.org/plosone/s/licenses-and-copyright`.

We require you to either (1) present written permission from the copyright holder to publish these figures specifically under the CC BY 4.0 license, or (2) remove the figures from your submission:

a. You may seek permission from the original copyright holder of Figures 3 and 4 to publish the content specifically under the CC BY 4.0 license.
We recommend that you contact the original copyright holder with the Content Permission Form (`http://journals.plos.org/plosone/s/file?id=7c09/content-permission-form.pdf`) and the following text: "I request permission for the open-access journal PLOS ONE to publish XXX under the Creative Commons Attribution License (CCAL) CC BY 4.0 (`http:`

//creativecommons.org/licenses/by/4.0/). Please be aware that this license allows unrestricted use and distribution, even commercially, by third parties. Please reply and provide explicit written permission to publish XXX under a CC BY license and complete the attached form."
Please upload the completed Content Permission Form or other proof of granted permissions as an ""Other"" file with your submission.
In the figure caption of the copyrighted figure, please include the following text: "Reprinted from [ref] under a CC BY license, with permission from [name of publisher], original copyright [original copyright year]."

b. If you are unable to obtain permission from the original copyright holder to publish these figures under the CC BY 4.0 license or if the copyright holder's requirements are incompatible with the CC BY 4.0 license, please either i) remove the figure or ii) supply a replacement figure that complies with the CC BY 4.0 license. Please check copyright information on all replacement figures and update the figure caption with source information. If applicable, please specify in the figure caption text when a figure is similar but not identical to the original image and is therefore for illustrative purposes only.

Figure 4 only consists of images that we took, and we have replaced the image of the clinical acquisition mobile pole in Figure 3 with a picture that we took. We have copyright permissions for both figures.

5. Please include captions for your Supporting Information files at the end of your manuscript, and update any in-text citations to match accordingly. Please see our Supporting Information guidelines for more information: http://journals.plos.org/plosone/s/supporting-information.

Thank you, we have included a caption for our supporting information.
